# Thoracoscopic esophagectomy with three-field lymphadenectomy for thoracic esophageal cancer in a patient with a double aortic arch: a report of a case

**DOI:** 10.1186/s40792-019-0640-7

**Published:** 2019-05-16

**Authors:** Hisashi Fujiwara, Takuji Sato, Naoya Okada, Takeo Fujita, Takashi Kojima, Hiroyuki Daiko

**Affiliations:** 1 Department of Esophageal Surgery, National Cancer Center Hospital East, 6-5-1, Kashiwa-no-ha, Kashiwa, Chiba, 277-8577 Japan; 2 Department of Esophageal Surgery, Tokyo Medical and Dental University, Tokyo, Japan; 3 Department of Esophageal Surgery, National Cancer Center Hospital, Tokyo, Japan; 4 Department of Gastrointestinal Oncology, National Cancer Center Hospital East, Kashiwa, Japan

**Keywords:** Double aortic arch, Vascular malformation, Vascular ring, Esophageal cancer, Esophagectomy, Thoracoscopy, Left thoracic approach, Preceding cervical procedure

## Abstract

**Background:**

We encountered an esophageal cancer patient with a double aortic arch (DAA) who underwent radical thoracoscopic esophagectomy with three-field lymph node dissection. A DAA generally makes it difficult to perform upper mediastinal lymph node dissection via both sides of the thoracic cavity. Furthermore, most patients with a DAA have a superior right aortic arch and right-sided descending aorta, which hampers radical esophagectomy with a typical right thoracic approach. We herein report our operative strategy of thoracoscopic esophagectomy via the left side of the thoracic cavity with a preceding cervical procedure.

**Case presentation:**

A 64-year-old man was diagnosed with esophageal squamous cell carcinoma in the upper esophagus at clinical Stage IIB (cT1bN1M0) according to the UICC-TNM classification 7th edition. First, we planned the preceding cervical procedure to complete upper mediastinal lymph node dissection, as the DAA prevented a bilateral thoracic approach to the upper mediastinum. We then planned the left thoracoscopic procedure to perform lymph node dissection below the left aortic arch, as the patient in our case had a right side-dominant DAA and right-sided descending aorta, as is common in such patients. We identified the bilateral recurrent laryngeal nerves during upper mediastinal lymph node dissection in the preceding cervical procedure and ultimately successfully resected the patient’s esophageal cancer.

**Conclusion:**

The cervical procedure preceding the left-thoracoscopic approach is reasonable for achieving radical esophagectomy for thoracic esophageal cancer in patients with a DAA.

## Background

A double aortic arch (DAA) is an extremely rare congenital vascular malformation caused by the remnant of the distal side of the right dorsal aorta. A DAA forms a complete vascular ring and is classified as a Stewart & Edwards type I vascular malformation [1, 2]. It often compresses the trachea and esophagus during infancy and childhood [3, 4]. Patients are therefore generally diagnosed with a DAA during this period due to symptoms of compression by a vascular ring or associated heart malformations. However, while it is often symptomatic during infancy, some cases occasionally have no symptoms and are only detected in adulthood by chance.

A DAA strongly affects surgery for esophageal cancer, which is used to manage the trachea, esophagus, and the surrounding tissue in the upper mediastinum from both sides of the thoracic cavity. Furthermore, most patients with a DAA have a dominant right aortic arch and right-sided descending aorta, which hampers radical esophagectomy with a typical right thoracic approach [5].

We herein report the case of an esophageal cancer patient with a DAA who successfully underwent curative esophagectomy with three-field lymph node dissection.

## Case Presentation

A 64-year-old man who had no symptoms was diagnosed with thoracic superficial esophageal cancer that was detected by screening upper endoscopy. He had a history of hypertension. He had also been found to have a vascular abnormality (DAA) as an adult and was observed in an asymptomatic state.

**Fig. 1:**
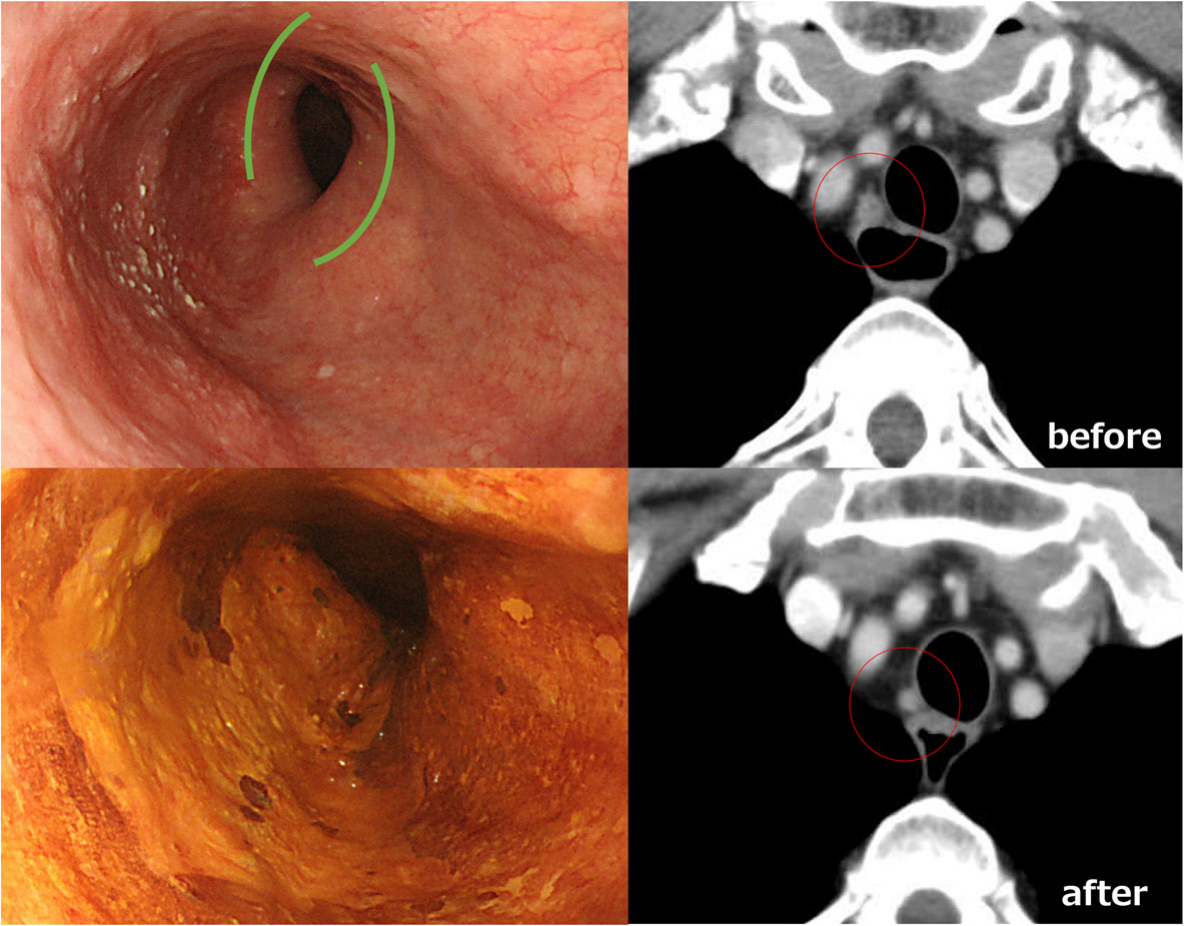
Endoscopic findings show the superficial esophageal squamous cell carcinoma at the left-side wall of the thoracic upper esophagus. Green lines indicate mild compression by the double aortic arch. Enhanced CT revealed the swelling of the right upper mediastinal lymph node before neoadjuvant chemotherapy and then the shrinking of this lymph node after chemotherapy

**Fig. 2:**
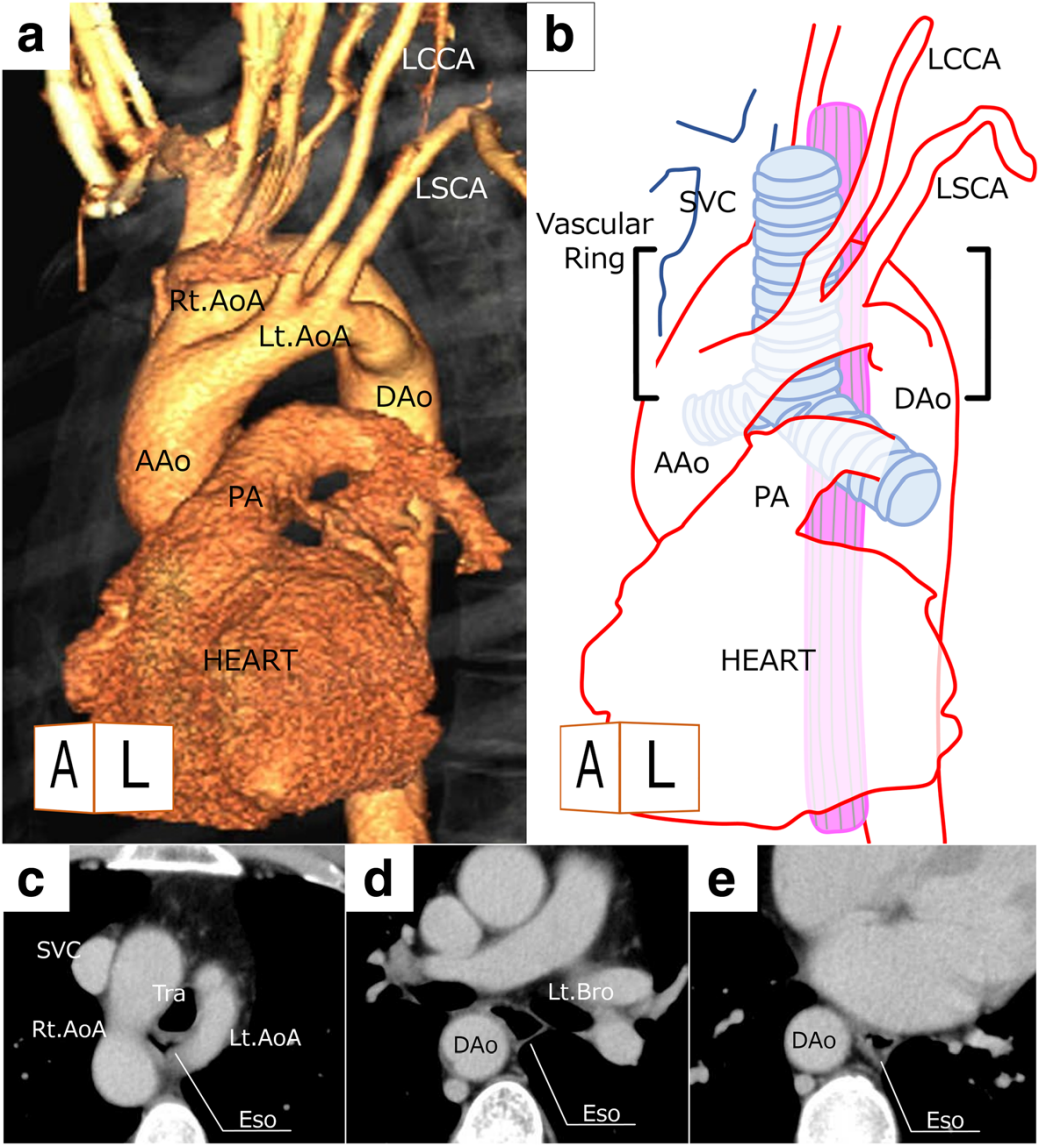
**a**, **b** Three-dimensional CT showed a double aortic arch. **c**–**e** Axial views on CT revealed the location of the esophagus and descending aorta. Approaching the esophagus from the right side of the thoracic cavity was expected to be difficult. AAo, ascending aorta; DAo, descending aorta; Eso, esophagus; Lt./Rt. AoA, left/right aortic arch; Lt. Bro, left bronchus; LCCA, left common carotid artery; LSCA, left subclavian artery; Tra, trachea

Physical examinations showed no unusual findings, and the laboratory examination data, including tumor markers, such as squamous cell carcinoma-related antigen and carcinoembryonic antigen, were all within normal ranges. Chest X-ray demonstrated a widening in the upper mediastinal silhouette, reflecting the superior right aortic arch. An endoscopic examination revealed superficial esophageal cancer located in the left side of the wall in the upper thoracic esophagus and the invasion of the submucosa (Fig. 1). A histological examination of biopsy specimens confirmed the presence of squamous cell carcinoma. Enhanced computed tomography showed a swollen lymph node in the right upper mediastinum, which was diagnosed as metastatic (Fig. 1). No distant metastasis was detected. Computed tomography also confirmed the DAA. The right aortic arch was dominant, and the descending aorta was located at the right side of the post-mediastinum, as is common in cases of DAA (Fig. 2). The patient was therefore diagnosed with upper thoracic esophageal cancer of cT1bN1M0 Stage IIB (UICC-TNM 7th) and a DAA.

He underwent neoadjuvant chemotherapy prior to sub-total esophagectomy with three-field lymphadenectomy. The neoadjuvant chemotherapy regimen was 2 courses of 5-FU (800 mg/m^2^) and cisplatin (80 mg/m^2^) every 3 weeks.

### Surgical Plan

We planned to perform radical subtotal esophagectomy with three-field lymph node dissection after neoadjuvant chemotherapy. We first planned to perform cervical procedure in a supine position before the thoracic procedure in order to identify the bilateral inferior laryngeal nerves and avoid causing them injury or inducing palsy. We also planned to perform upper mediastinal lymph node dissection during this preceding procedure because the DAA was expected to interfere with upper mediastinal dissection attempted via either side of a transthoracic approach. We then planned to perform lymph node dissection via a left-thoracoscopic approach below the left aortic arch, as we worried that the right-sided descending aorta might interfere with a right-thoracic approach (Fig. 2). The laparoscopic procedure was planned to be performed via an abdominal procedure in a supine position. Reconstruction would use the gastric tube pulled up via the retrosternal route with cervical esophago-gastric anastomosis.

### Intraoperative and postoperative findings

**Fig. 3:**
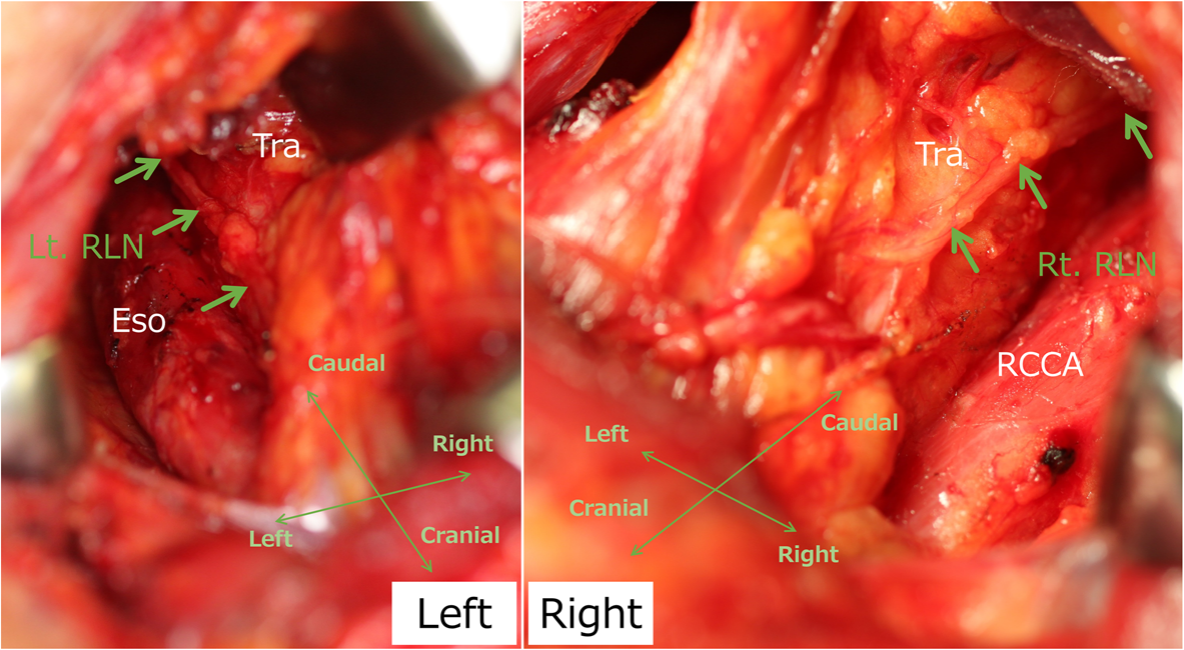
Intraoperative findings of trans-cervical upper mediastinal lymph node dissection in the preceding cervical procedure. Green arrows indicate the recurrent laryngeal nerves. We were able to identify the bilateral recurrent laryngeal nerves ascending along the trachea and esophagus just like the typical left recurrent laryngeal nerve. Eso, esophagus; Lt./Rt. RLN, left/right recurrent laryngeal nerve; Tra, trachea

**Fig. 4.**
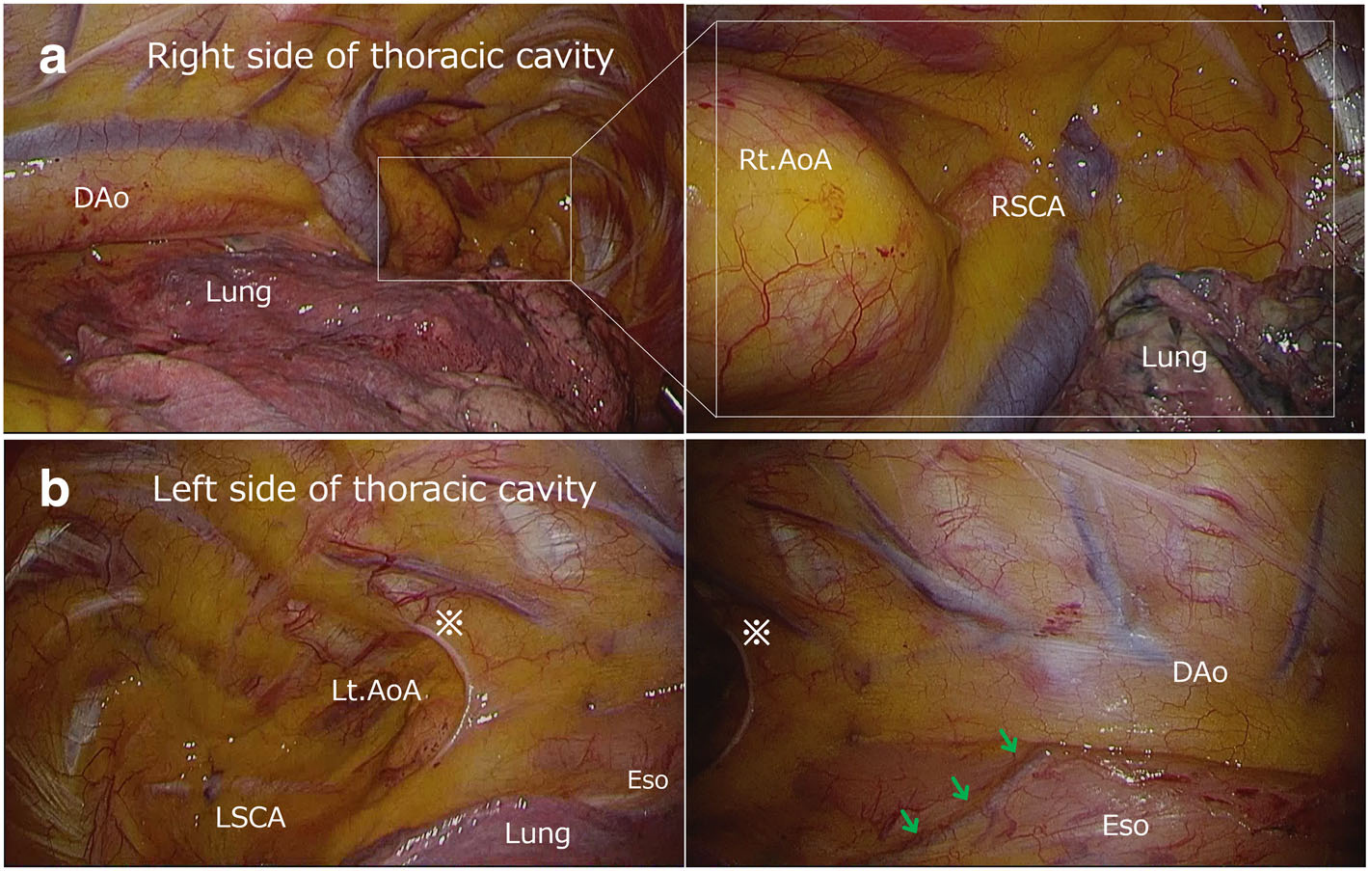
**a** Intraoperative findings from the right side of the thoracic cavity. The superior right aortic arch and right side of the descending aorta obscured the esophagus and interfered with the normal right thoracic approach. **b** Intraoperative findings from the left side of the thoracic cavity. We were able to approach the esophagus just below the left aortic arch but could not approach the upper mediastinum or the right side. Postmediastinal reconstruction seemed impossible. Green arrows indicate the left bronchial artery. DAo, descending aorta; Eso, esophagus; Lt./Rt. AoA, left/right aortic arch; LSCA/RSCA, left/right subclavian artery

**Fig. 5:**
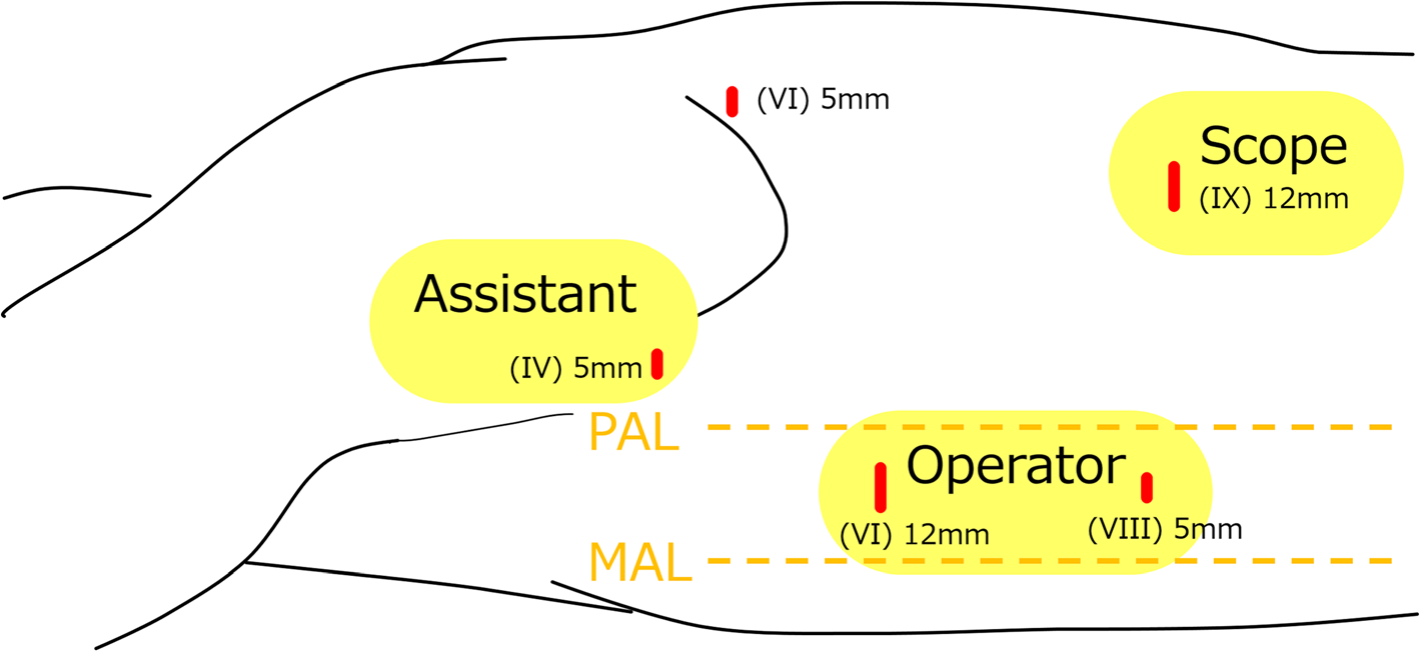
This figure shows the port position during the left thoracoscopic procedure, with the number of intercostal spaces and the diameter of each inserted port. The operator, assistant, and scopist used each port, as shown in this figure. MAL, middle axillary line; PAL, posterior axillary line

In the preceding cervical procedure performed in a supine position, we identified the bilateral inferior laryngeal nerves, which were thought to be recurrent at each side of the aortic arch (Fig. 3). After upper mediastinal dissection was performed, the left thoracoscopic procedure in a prone position was performed for middle and lower mediastinal lymph node dissection below the left aortic arch. We first confirmed that the right-sided aortic arch and descending aorta would interfere with the usual right thoracic approach (Fig. 4a). Upper mediastinum dissection was also deemed impossible via a bilateral thoracic approach because of the bilateral aortic arches and subclavian arteries, as expected preoperatively (Fig. 4a, b). Postmediastinal reconstruction also seemed impossible. The port position for the left thoracoscopic procedure was set symmetrically to our normal right thoracoscopic procedure for middle to lower mediastinal dissection, as shown in Fig. 5. No major anatomical findings other than those noted preoperatively were observed during the left thoracoscopic procedure. We were unable to identify where the thoracic duct ascended because of the preservation of the thoracic duct. We were also unable to confirm the details concerning both recurrent laryngeal nerves around each aortic arch.

The abdominal procedure in a supine position was performed laparoscopically with the simultaneous cervical procedure for bilateral supraclavicular lymph node dissection. Reconstruction was performed with cervical esophago-gastric tube anastomosis. The gastric tube was pulled up through a retrosternal route as planned. Three-field lymph node dissection and complete resection (R0) were achieved. The operative time was 8 h 9 min, and the total bleeding was 70 ml. No vocal cord palsy was observed on flexible laryngoscopy after the operation.

The patient’s postoperative course included minor leakage that was cured conservatively after 2 weeks, and he was discharged at postoperative day 29. The pathological diagnosis was ypT1bN0M0 Stage IA (UICC-TNM 7th edition). The patient was followed for 2 years with no signs of cancer recurrence.

## Discussion

In this report, we emphasize the usefulness of the cervical procedure preceding esophagectomy via the left thoracoscopic approach for thoracic esophageal cancer in a patient with a DAA. Through this approach, we successfully performed radical esophagectomy with three-field lymph node dissection for thoracic esophageal cancer in a patient with a DAA, with outcomes nearly the same as those in patients with a normal aortic formation.

Five surgical cases of esophagectomy in a patient with a DAA have previously been reported, and the details of these cases as well as our own are summarized in Table 1 [6–10]. Each surgical approach differed, although the anatomical features of the cases were markedly similar. Our approach combining a preceding cervical procedure and left thoracoscopic approach appears to be unique. Furthermore, our case is the first in which thoracoscopic esophagectomy was performed in a prone position.

**Table 1:** Summary of the past five case reports and our report of esophagectomy in patients with a double aortic arch

	2011, Matono [6]	2012, Kubo [7]	2015, Uemura [8]	2018, Peng [9]	2018, Clement [10]	Our case
Age/sex	50 years old/male	70 years old/male	63 years old/male	65 years old/male	57 years old/male	64 years old/man
Tumor location	Mt, type 2	Mt, type 0-Is	Lt, type 2	UtMt	Unknown	Ut, type 0-IIc
Anatomical features						
Aortic arch	Right dominant	Right dominant	Right dominant	Right dominant	Right dominant	Right dominant
Descending aorta	Right side	Right side	Right side	Right side	Right side	Right side
Operation/approach						
Preceding cervical procedure	Yes	No	Yes	No	No	Yes
Thoracic procedure	Right thoracotomy	Left thoracotomy	Right thoracotomy	Left thoracotomy	Left thoracosbdominal	Left thoracoscopy
Abdominal procedure	Hand-assisted laparoscopy	Unknown	Unknown	Unknown		Laparoscopy
Reconstruction	Gastric tube	Gastric tube	Gastric tube	Gastric tube	Unknown	Gastric tube
	Retrosternal route	Retrosternal route	Retrosternal route	Postmediastinal route		Retrosternal route
Diagnosis	cT3	Unknown	ypT3N1M0 Stage IIIA	cT1bN0M0	Unknown	cT1bN1M0 Stage IIB
			(UICC-TNM 7th)	(UICC-TNM 7th)		(UICC-TNM 7th)
Prognosis	Dead	Unknown	Unknown	Unknown	Unknown	Alive (2 years)

A cervical procedure preceding esophagectomy is useful for performing appropriate upper mediastinal lymph node dissection, and it can help to preserve the bilateral inferior laryngeal nerves, such as in cases associated with an aberrant right subclavian artery. The tumor in our patient was superficial, and the preoperative examination showed no evidence of multiple lymph node metastasis; therefore, invasive sternotomy was not necessary. In cases with multiple metastatic upper mediastinal lymph nodes, we might add sternotomy to the cervical procedure in order to perform radical upper mediastinal lymph node dissection.

The left thoracic approach was deemed reasonable for facilitating the visualization of the esophagus in a case with a right-sided descending aorta. However, the heart’s location in the left thorax interrupted the procedure of middle to lower mediastinal dissection, in contrast to a typical right thoracic approach. The left thoracoscopic approach in a prone position had an advantage of minimizing the interference of the heart in the left thorax compared to left thoracotomy in a right lateral decubitus position. Thoracoscopy and laparoscopy absolutely helped minimize the wound and surgical invasiveness. I think that a trans-hiatal (laparoscopic) approach may be a promising alternative to a left-thoracoscopic approach as long as upper mediastinal lymphadenectomy is performed during the cervical procedure [11].

**Fig. 6:**
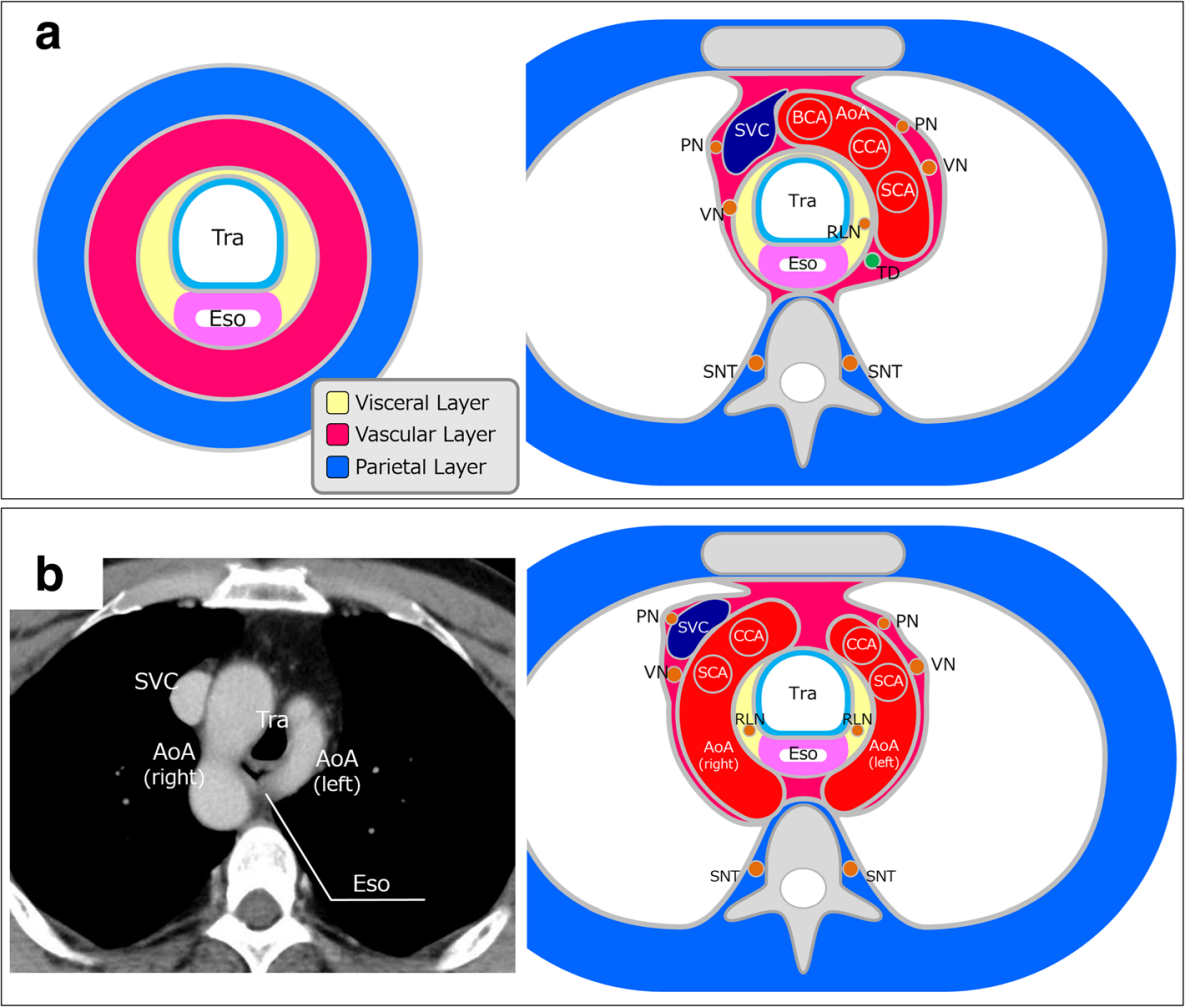
**a** A concentric-structured model and surgical anatomy concerning the layer structure in the normal upper mediastinum. This anatomical model is based on human embryonic development. This is the essence of our surgical concept and practical anatomy. These figures are reproduced with permission from Fujiwara H, Kanamori J, Nakajima Y, Kawano T, Miura A, Fujita T, et al. An anatomical hypothesis: a “concentric-structured model” for the theoretical understanding of the surgical anatomy in the upper mediastinum required for esophagectomy with radical mediastinal lymph node dissection. Diseases of the esophagus: official journal of the International Society for Diseases of the Esophagus 2018 (Epub ahead of print) [12]. **b** The surgical anatomy of the upper mediastinum in patients with a double aortic arch, which we developed based on the concentric-structured model. We planned and performed the surgical procedure in this rare case while keeping in mind this theoretical surgical anatomy. AoA, aortic arch; BCA, brachiocephalic artery; CCA, common carotid artery; Eso, esophagus; PN, phrenic nerve; RLN, recurrent laryngeal nerve; SCA, subclavian artery; SNT, sympathetic nerve trunk; SVC, superior vena cava; TD, thoracic duct; Tra, trachea; VN, vagal nerve

Disorientation concerning the surgical anatomy before and during surgery was expected, as a DAA is a rare vascular malformation. However, engaging in embryology-based thinking made it easier to understand the surgical anatomy, even in this patient with a rare vascular malformation. We developed a new hypothetic surgical anatomy model, named the “concentric-structured model,” which was based on the concept of human embryonic development, particularly with regard to the development of the aortic arch system (Fig. 6a) [12]. This hypothesis may facilitate the theoretical understanding among esophageal surgeons of the surgical anatomy of the upper mediastinum in cases with vascular anomalies, as well as typical cases. We preoperatively planned the surgical procedures with reference to the surgical anatomy derived from this concentric-structure model (Fig. 6b). Embryology-based thinking can facilitate the understanding of the course of the inferior laryngeal nerves (recurrent laryngeal nerves) in rare cases with a DAA. We suspect that considering the surgical anatomy based on the development of the aortic arch system may also support the safe performance of esophagectomy in other rare cases with vascular malformations.

## Conclusion

We encountered a patient with thoracic esophageal cancer and a double aortic arch. We performed a cervical procedure preceding esophagectomy with a left thoracoscopic approach and ultimately achieved curative resection and three-field lymph node dissection with minimal surgical invasiveness.

## Abbreviations

5-FU: 5-Fluorouracil
DAA: Double aortic arch
UICC: Union for International Cancer Control
